# Pathological risk-propensity typifies Mafia members’ cognitive profile

**DOI:** 10.1038/s41598-020-65486-z

**Published:** 2020-05-22

**Authors:** Gerardo Salvato, Maria Laura Fiorina, Gabriele De Maio, Elisa Francescon, Daniela Ovadia, Luisa Bernardinelli, Amedeo Santosuosso, Eraldo Paulesu, Gabriella Bottini

**Affiliations:** 1 Department of Brain and Behavioral Sciences, University of Pavia, Pavia, Italy; 2 Cognitive Neuropsychology Centre, ASST “Grande Ospedale Metropolitano” Niguarda, Milano, Italy; 3 NeuroMi, Milan Center for Neuroscience, Milan, Italy; 4 European Centre for Law, Science and New Technologies (ECLT), University of Pavia, Pavia, Italy; 5 Psychology Department and NeuroMI-Milan Center for Neuroscience, University of Milano-Bicocca, Milan, Italy; 6 fMRI Unit, IRCCS Istituto Ortopedico Galeazzi, Milan, Italy

**Keywords:** Cognitive neuroscience, Human behaviour

## Abstract

Since the recruitment process, Italian Mafias impose on their members a strict code of conduct. These rigid rules regulate their private and public behavior, implying a total adhesion to the group’s values. Such juridical and social aspects substantially distinguish organized crime (OC) from ordinary crime. It is still unknown whether these two categories of offenders also show distinctive cognitive traits. Here we investigated the frontal lobe cognitive functions of 50 OC prisoners from the Mafia and 50 non-OC prisoners based on the performance of 50 non-prisoner controls. We found that OC members were more likely to show pathological risk-propensity than non-OC prisoners. We interpret this finding as the result of the internal dynamics of Mafia groups. OC is a worldwide threat, and the identification of cognitive traits behind criminal behavior will help in devising focused prevention policies.

## Introduction

Organized crime (OC) groups are very stable organizations governed by a complex hierarchical structure and bounded by rigorous norms^1,2^. OC recruits specific individuals on whom they impose a veritable status contract^3^. In the case of archetypical organizations such as the Italian Mafias, through the ritual initiation, novices are required to assume a new permanent identity as “men of honor”^3–5^. As such, OC members regulate their behavior according to canons of masculinity, respect, and secrecy, to increase their reputation and preserve their position within their group^3,6^. Their criminal activities, as well as their private lives, are regulated by codes of conduct known as commandments^1^, which are shared norms that build a strong collective identity, enhancing the group’s cohesion. Indeed, OC activities implicate a close interaction between co-offenders and firm adherence to the group’s values. These peculiar features substantially distinguish OC from ordinary crime (non-OC) from both a juridical and social perspective, leaving an open question concerning possible distinctions between offender types, including at the behavioral level. One might suppose that distinctive features of OC members’ behavioral profiles emerge from cognitive variations in frontal lobe executive functions, i.e., high-order goal-oriented processes controlling flexible behaviors to support a proficient interaction with the environment^7,8^. Previous studies have shown that executive dysfunctions are predictive of specific criminal behaviors^9,10^. So far, cognitive neuroscience has provided evidence for selective executive deficits in non-OC offenders, which include planning strategies^11^ and working memory^12^. However, it is currently unknown whether there is also a pattern of executive (dys)functioning that is characteristic of OC members. Clear-cut experimental evidence on this topic is scarce. Here, we were able to test these hypotheses and assessed 50 Mafia-type OC prisoners to explore as comprehensively as possible the various facets of executive functions. On the basis of the most recent neurocognitive models of frontal lobe functioning^7,8^, we used a series of computerized cognitive tasks investigating planning, working memory, risk-taking behavior, flexibility, inhibition, and sustained attention. By comparison, the same tests were administered to 50 ordinary non-OC prisoners, and 50 age-gender matched non-prisoners. We hypothesized that OC members, as part of an organized and pervasive group with its specific group dynamics, may show distinctive cognitive traits compared to non-OC prisoners. For instance, it is increasingly recognized that self-identification with a group modulates frontal lobe functions^13–15^. More specifically, the affiliation to a group that endangers the lives of its affiliated “men of honor” could boost risk-taking behavior.

## Results

As a first step, we compared the socio-demographic and psychological variables between the two groups of prisoners to detect possible differences, and eventually to adjust subsequent analysis for the observed effect. The OC and non-OC groups did not differ in age (*t*_(98)_ = −0.87; *p* = 0.382), education (*t*_(98)_ = 0.38; *p* = 0.706), global cognitive functioning (*t*_(98)_ = −1.26; *p* = 0.212), fluid intelligence (*t*_(98)_ = 0.08; *p* = 0.940), anxiety (*t*_(98)_ = −0.47; *p* = 0.642), depression (*t*_(98)_ = −1.03; *p* = 0.307), psychopathy personality traits (*t*_(98)_ = 0.20; *p* = 0.844), and number of incarcerations (*t*_(98)_ = −0.38; *p* = 0.704). Nevertheless, at the time of testing, they differed in periods of detention (*t*_(98)_ = −5.58; *p* < 0.001): OC offenders had been staying in prison on average for 12.7 years (*SD* = 9.2), while non-OC prisoners were incarcerated on average for 4.9 years (*SD* = 3.5) (Table 1). Such a difference was expected because of the Italian Law System: for the same crime committed, OC members received a harsher sentence than non-OC individuals, because they are also convicted of Mafia association. We considered this variable as a covariate in the subsequent analyses.

**Table 1:** Demographic data, global cognitive functions, and psychological assessment.

	**Organized Crime** prisoners (N = 50) *Mean [95% CI]*	**Non-Organized Crime** prisoners (N = 50) *Mean [95% CI]*	*p-value*
Age	48.7 [45.8, 51.6]	46.6 [42.8, 50.4]	0.382
Education	10.7 [9.8, 11.5]	10.9 [9.9, 11.8]	0.706
Global cognitive Functioning	92.7 [91.4, 94]	91.4 [89.8, 93.1]	0.212
Fluid Intelligence	30.9 [29.8, 32.2]	30.9 [29.9, 31.9]	0.940
Anxiety	42.4 [38.9, 45.8]	40.8 [37.2, 44.3]	0.642
Depression	15.9 [12.9, 19]	13.9 [11.4, 16.4]	0.307
Psychopathy personality traits	273.2 [264.7, 281.8]	274.5 [265.1, 283.8]	0.844
Number of incarcerations	2.6 [2.1, 3.1]	2.5 [1.8, 3.1]	0.704
Imprisoning time (years)	12.7 [10.9, 15.33]	4.9 [3.9, 5.9]	<0.001*

As a second step, we standardized the OC and non-OC participants’ test scores based on the non-prisoner control group’s values. Scores from the two groups of prisoners were transformed in z-scores according to the mean and standard deviation calculated for each test using the control group scores. This procedure allowed us to compare different tests and gauge possible cognitive deficits (scores < or > 2 standard deviations^16,17^). As each test provided different outcome variables (e.g., reaction times, false alarms, error percentage, and others), we applied a data-driven approach. To select one variable of interest for each executive function subcomponent, we used multiple Partial Least Square Regressions. Lastly, we performed a Binary Logistic Regression to determine domains associated with group membership. This model included Group (OC, non-OC) as a dependent variable, and we inserted the scores from the Stockings of Cambridge test (planning), Spatial Working Memory task (working memory), Balloon Analogue Risk Task (BART) and the Body Analogue Risk Task (BoART) (risk-taking behavior associated with non-biological and biological stimuli), Multitasking Test (flexibility and inhibition), and Rapid Visual Information Processing test (sustained attention) as predictors (see the methods section for a detailed description of the tests of each specific function). The final model was significant (omnibus test χ2_(2df)_ = 36.1; *p* < 0.001; Hosmer and Lemeshow χ2_(8df)_ = 4.5; *p* = 0.805)), and it explained 40.7% of Nagelkerke’s pseudo-variance^18^. We found that the odds of an OC prisoner presenting with impaired risk-taking behavior (elevated risk-taking behavior, e.g., scores >2 standard deviations of the controls’ mean) were 6.4 times greater than the odds of a non-OC prisoner (Wald χ2_(1)_ = 5.5; *p* = 0.019; *OR* = 6.44; *CI* = 1.36–30.5); the other variables were removed from the model (all *ps* > 0.05) (Table 2). A Receiver Operating Characteristic (ROC) curve was calculated to test whether the predicted probability of the model successfully discriminated OC from non-OC. The results showed an accuracy (area under the curve) of 0.80 (*CI*: 0.71, 0.89; *p* < 0.001). Lastly, as it has been shown that risk-taking behavior may be associated with personality traits^19^, we ran an additional analysis correlating the risk index scores of the two prisoners’ groups with the psychopathic personality traits test scores (Psychopathic Personality Inventory-Revised (PPI-R))^20^. We found no evidence for a correlation between risk-taking behavior and the eight PPI-R subscales (α level = 0.006; *Bonferroni* corrected).

**Table 2:** Regression analysis results.

**Predictors**	**Wald**	**Sig**.	**Exp(B)**	**CI lower**	**CI Upper**
***variables in the equation***					
Imprisoning time (years)	17.1	0.000	1.2	1.1	1.4
Risk-taking (BART)	5.5	0.019	6.4	1.4	30.5
***variables not in the equation***					
Cognitive Flexibility		0.542			
Spatial Working Memory		0.162			
Sustained Attention		0.733			
Risk-taking (BoART)		0.365			
Planning		0.097			

## Discussions

These findings demonstrate that affiliation to pervasive groups such as the Mafias influences their members’ behavior. This is consistent with social psychology studies showing that group dynamics modulate individual behavior in a pervasive way. People with a strong identification to the group they belong to behave differently from people who perceive themselves as isolated individuals. Interestingly, the main cognitive dysfunction characterizing OC members is a pathological risk-taking behavior, whereas the other predictors were not significant. Such a cognitive feature did not correlate with psychopathy traits.

Typically, the group pushes the individuals towards more risky behavior, a tendency labeled as “risk-shift phenomenon”^15^. In a seminal study, Wallach and colleagues^21^ showed that when group interaction occurs, people tend to make riskier decisions compared to those made individually. In their study, two groups of subjects were asked to individually decide on 12 everyday life situations with increasing risk/reward trade-offs (choice dilemma procedure). Furthermore, the experimental group was asked to discuss to reach a consensual decision. In this case, subjects showed an overall general shift towards riskier decisions that persisted for quite a long period (2 to 6 weeks). These results have been replicated several times with different paradigm variations^22–24^. Two hypotheses have been proposed to explain this phenomenon: i) shared/spreading of responsibility in the group, so that risky decisions are perceived as more affordable, ii) persuasion exerted by the most influential individuals, usually high-risk takers, and perceived as holders of greater forcefulness by the group. Reframing these hypotheses into the OC background, OC offenders may show higher propensity towards risky behavior as they share responsibility with the other members of the group. Furthermore, OC members may feel protected by their criminal network, whereas non-OC offenders act as lone wolves. Nevertheless, we cannot exclude the second hypothesis as, in our study, we exclusively tested individuals with a low hierarchical standing, merely crime executors, and typically, Mafia bosses are considered untouchable, highly influential leaders whose decisions are not to be disputed. However, the risky-leader theory has been criticized by Hoyt and Stoner^24^ as they replicated the risk-shift effect while neutralizing any leadership effects of highly risk-prone persons. In this case, group discussion to consensus still produced decisions that were riskier than the mean of the individual decisions of group members. Furthermore, the risk-shift phenomenon does not exclusively occur when the group can discuss the decisions to be taken consensually, as it has been demonstrated that this effect is also obtained in the absence of this interaction^25^. We also propose a third hypothesis that concerns the “man of honor” status, which is a crucial and symbolic dimension of Mafia organizations. To maintain this status, OC members should continuously demonstrate their willingness to do anything, including criminal acts. Such a philosophy of life inevitably leads to pathological risk-taking behavior that paradoxically protects OC members from unwanted changes of status within the group. Interestingly, contrary to previous experimental paradigms, the test that was used to evaluate risk-taking behavior, the Balloon Analogue Risk Task (BART)^26^, models real-world risk behavior through the conceptual frame of balancing the potential for reward versus loss. In brief, during the BART, participants are offered the chance to virtually earn money by pumping the balloon up by clicking a keyboard button. Each click causes the balloon to incrementally inflate and virtual money to be added to a counter up until some threshold, at which point the balloon is overinflated and explodes. Each pump entails greater risk, but also greater potential reward. If the participants choose to cash-out before the balloon explodes, then they virtually collect the money earned for that trial, but in case the balloon explodes, earnings for that trial are lost. Importantly, this test provides a highly generalizable outcome as it correlates with several real-world risky behaviors, for example, drug abuse^27^, gambling, risky sexual behavior^28^, smoking^29^, and substance abuse with coexisting conduct problems in adolescent patients^30^.

In conclusion, the current study shows that OC members are more likely to show pathological risk propensity compared to non-OC offenders. Group membership appears to be an important dimension of this pathological attitude. This executive dysfunction should be taken into account together with innate and environmental components, both of which are required for a cognitive trait to develop^31,32^. More information concerning the etiology of the OC-membership might be useful in future studies to better understand the risk-taking behavior of those individuals who were born into the OC-family compared to those who came from outside the OC structures. OC is a worldwide threat, and understanding the dynamic interplay between all these components could help devise effective prevention policies. So far, several prevention programs have been devised to foster the development of emotional and social skills already in childhood and adolescence in case of dysfunctional behaviors. In particular, the Social and Emotional Learning (SEL) programs proved effective in promoting such skills^36–38^. The Collaborative to Advance Social and Emotional Learning (CASEL)^39^ has developed a framework of key SEL skills, including self-awareness, self-management, social awareness, relational skills, and responsible decision-making. The latter, is particularly relevant to the case of OC as it fosters the ability to make decisions based on consideration of ethical standards, safety concerns, appropriate standards of conduct, respect for others, and likely consequences of various actions. These conclusions underpin the rationale for the adoption of educational and social intervention programs (e.g., the SEL) that may be particularly effective in specific “at-risk areas” in which mafia and other forms of organized crime are present^40,41^. We anticipate that the promotion of more controlled decision-making skills in young adults may reduce their future recruitment into the local OC group. As it is well known, the translation of a proof of principle into educational policies and programs requires a long and laborious process and vast resources (http://www.oecd.org/education/implementing-policies/).

### Limitations

This study has some limitations. For instance, we used a control matched non-prisoners control group to determine normative scores for each computerized test. We acknowledge that a group of 50 individuals could not be an adequate proxy for norms in the general population. It is also important to bear in mind that although risk-taking behavior was the only pathological trait distinguishing between the two groups of prisoners, this result did not imply that the two groups were different with respect to other/different cognitive functions. Thus, it is possible that the two prisoners’ groups showed equal cognitive functioning (or dysfunctioning) at planning, spatial working memory, flexibility and inhibition, and sustained attention. According to our experimental question, our statistical approach explored the hypothesis of a pathological cognitive function predicting the OC membership. Nonetheless, future studies may be specifically conceived to test differences in the cognitive functioning of OC members compared to other types of prisoners. So far, only one study has explored the neuropsychological profile of OC prisoners, who were, in that case, all affiliated to a drug trafficking Mexican cartel^33^. Ostrosky and collaborators^33^ have compared this type of OC offenders with a group of non-prisoner healthy individuals on executive functions. They have found differences in several cognitive functions, including planning, working memory, and inhibition. Unfortunately, the provided results are hardly generalizable because the only comparison between prisoners and non-prisoners left open the possibility that those differences may be the result of the condition of detention *per se*, rather than belonging to the drug trafficking OC. Moreover, in the same study, the inmate group was divided into five subcategories (kingpins, money launderers, protectors, enforcers, and distributors/producers). Such categories were strongly unbalanced by sample size and compared with each other, possibly affecting the statistical power and the Type I error rates.

## Materials and methods

### Participants

According to *a priori* power analysis (see below), one hundred male inmates were randomly selected from two lists provided by two Italian Penitentiaries comprising respectively 1125 and 1253 prisoners. To be included in the organized crime (OC) group, prisoners had to have committed a Mafia-related crime (article *416 bis* of the Italian Penal Code). We tested 50 OC prisoners and 50 non-organized crime prisoners (non-OC). The OC and non-OC groups were balanced according to the nature of the crime committed (25 violent and 25 nonviolent prisoners per group). We also recruited a matched control group, including 50 non-prisoner participants, which allowed us to determine the performance at the experimental computerized tasks in a non-incarcerated population. The three groups of participants were matched for age, gender, and years of education. All participants were native Italian speakers and had a normal or corrected-to-normal vision. Participants had no history of neurological or psychiatric disorders. Informed consent was obtained before participation in the experiment using a form purposely developed for this project within the framework of the Charter of Fundamental Rights of the European Union and the principles of the Office for Human Research Protection of the US Department of Health and Human Services for research on prisoners. In accordance with the Declaration of Helsinki (BMJ 1991; 302: 1194), all the experimental procedures were approved by the Ethical Committee of the Department of Brain and Behavioral Sciences, University of Pavia (protocol No. 010).

### Sample size calculation

Studies investigating cognitive functions in OC compared to non-OC prisoners are absent. For this reason, we estimated the group size needed to show a between-group difference in executive functioning by using a study in which frontal lobe functions had been tested in a group of non-OC prisoners. Dolan and colleagues^34^ tested memory and executive functions comparing prisoners convicted for violent crimes to healthy controls. The group of prisoners showed a mean Executive Function Index score of 2.16 (*SD* = 3.9), whereas the healthy controls had a mean of 5.57 (*SD* = 2.3). Based on these data, we hypothesized that one of the groups in our study would show an executive functions deficit with an *alpha* = 0.05 on an independent means two-tailed *t*-test. Using a freely available sample-size calculating tool (G*Power), we determined a suggested sample size of 25 participants per group. Although in our study we were not interested in differences between violent and non-violent offenders, we nevertheless balanced the two groups (OC, non-OC) of prisoners for the type of crime committed to deal with a possible confounding due to the differences between violent and nonviolent prisoners on the executive function tests that we used here (see for instance^35^). More precisely, we tested 50 criminals per group (OC, non-OC), considering in each group 25 violent and 25 nonviolent prisoners.

### Neuropsychological assessment

#### Cognitive screening

To exclude that possible general cognitive impairment or intelligence deficit could influence the performance in the other tests, we administered two neuropsychological tests assessing the integrity of their global cognitive functioning and intelligence: the Addenbrooke’s Cognitive Examination-Revised (ACE-R)^36^ and the Raven’s Colored Progressive Matrices (CPM)^37^. ACE-R is a brief battery that provides an evaluation of six cognitive domains (orientation, attention, memory, verbal fluency, language, and visuospatial ability). The total score indicated the global cognitive functioning clinical status. CPM is a culture-free test for nonverbal intelligence. It consists of 36 nonrepresentational colored design patterns incomplete in the bottom right corner. Participants were required to select the best completion pattern from among six alternatives. The available normative scores for the Italian population^37,38^ were used to diagnose possible neuropsychological deficits. All the participants showed preserved global cognitive functioning and fluid intelligence.

#### Mood

We also tested anxiety and depression using the State-Trait Anxiety Inventory (STAI)^39^ and the Beck Depression Inventory (BDI)^40^. The STAI is a psychological inventory based on a 4-point Likert scale and consists of 40 questions on a self-report basis. The STAI measures two types of anxiety – state anxiety, or anxiety about an event, and trait anxiety, or anxiety level as a personal characteristic. The BDI is a 21-question multiple-choice self-report inventory, one of the most widely used psychometric tests for measuring the severity of depression.

#### Psychopathy

Lastly, we assessed psychopathic personality traits using the Psychopathic Personality Inventory-Revised (PPI-R)^20^. The PPI-R is a personality test for traits associated with psychopathy in adults. It consists of a series of statements to which subjects respond, indicating how accurately the statement describes them using a 4-point Likert scale (“False,” “Mostly False,” “Mostly True,” “True”). The PPI-R scores are grouped into eight subscales, seven of which can be organized into two higher-order factors: Fearless Dominance, including the subscales Stress Immunity, Social Influence, and Fearlessness; and Self- Centered Impulsivity, including the subscales Rebellious Nonconformity, Blame Externalization, Machiavellian Egocentricity, and Carefree Nonplanfulness.

#### Experimental tasks

Following the neuropsychological screening, seven tests from the Cambridge Automated Neuropsychological Test Battery (CANTAB; Cambridge Cognition 2006; 2008) were used to assess the frontal lobe profile of our participants. Extensive descriptions and interactive demonstrations of the tasks are available on the website of the manufacturer (www.cambridgecognition.com). The tests were administered using a 12.1-in. touch-screen tablet (screen resolution of 1280 × 800). We also used a newly developed paradigm to assess risk-taking behavior with biological and non-biological stimuli. The following tasks were administered:

#### Cognitive flexibility and inhibition

The Multitasking Test explored the participant’s ability to manage conflicting information provided by the direction of an arrow and its location on the screen and to ignore task-irrelevant information. The test displayed an arrow, which could appear on either side of the screen (right or left) and could point in either horizontal direction (to the right or the left). Each trial displayed a cue at the top of the screen that indicated to the participants whether they had to select the right or left button according to the “side on which the arrow appeared” or the “direction in which the arrow was pointing.” In some sections of the task, this rule was consistent across trials (single task), while in others, the rule was allowed to change from trial to trial in a randomized fashion (multitasking). Flexibly using both rules places a higher demand on cognition than using a single rule. Some trials displayed congruent stimuli (e.g., an arrow on the right side pointing to the right). In contrast, other trials displayed incongruent stimuli (e.g., an arrow on the right side of the screen pointing to the left), which required a higher cognitive demand. As outcome variables, we used the following:

(i) Multitasking incongruency cost (median): The difference between the median latency of response (from stimulus appearance to button press) on the trials that were congruent versus the incongruent trials. This was calculated by subtracting the median congruent-trial latency (in ms) from the median incongruent-trial latency. A positive score indicates that the subject was faster on congruent trials, and a negative score indicates that the subject was faster on incongruent trials. A higher incongruency cost indicates that the subject took longer to process conflicting information.
(ii) Multitasking cost (median): The difference between the median latency of response (from stimulus appearance to button press) during assessed blocks in which both rules were used versus assessed blocks in which only a single rule was used. Calculated by subtracting the median latency of response during single-task blocks from the median latency of response during multitasking blocks. A positive score indicates that the subject responded more slowly during multitasking blocks and indicates a higher cost of managing multiple sources of information.
(iii) Reaction latency (median): The median latency of response (from stimulus appearance to button press). Calculated across all correct trials.
(iv) Total incorrect: The number of trials for which the outcome was an incorrect response (subject pressed the wrong button within the response window). Calculated across all assessed trials.

#### Planning

The Stockings of Cambridge (SOC) is a computerized modified version of the Tower of London test and assessed the participants’ ability to engage in planning and spatial problem-solving. Participants were presented with a horizontally split screen, and verbally instructed to move the colored balls in the lower half to copy the pattern of colored balls in the upper half. Difficulty slowly increased from requiring a minimum of two moves to requiring a minimum of five moves. As outcome variables, we used the following:

(i) Median Latency to First Choice: The median latency, measured from the appearance of the stocking balls until the first box choice was made by the subject. Calculated across all assessed trials where the subject’s first response was correct.
(ii) Problems solved on the first choice: The total number of assessed trials where the subject chose the correct answer on their first attempt. Calculated across all assessed trials.

#### Sustained attention

The Rapid Visual Information Processing (RVP) test was used to measure sustained attention. In this task, participants were required to detect target sequences of digits displayed on the side of the screen (for example, 2-4-6, 3-5-7, 4-6-8). A white box was shown in the center of the screen, inside of which digits from 2 to 9 appeared in a pseudo-random order, at the rate of 100 digits per minute. When the participant saw the target sequence, they responded by selecting the button in the center of the screen as quickly as possible. The level of difficulty varied, with the participant having to watch for either one- or three-target sequences at the same time. As outcome variables, we used the following:

(i) A′ (A prime) is the signal detection measure of a subject’s sensitivity to the target sequence (a string of three numbers), regardless of response tendency (the expected range is 0.00 to 1.00; bad to good). In essence, this metric is a measure of how good the subject is at detecting target sequences.
(ii) Median response latency: The median response latency on trials where the subject responded correctly, calculated across all assessed trials.
(iii) Probability of false alarm: The number of sequence presentations that were false alarms divided by the number of sequence presentations that were false alarms plus the number of sequence presentations that were correct rejections: (false alarms ÷ (false alarms + correct rejections)).

#### Working memory

The Spatial Working Memory task (SWM) was used to measure working memory. This task assesses the participants’ ability to retain spatial information and to manipulate remembered items in working memory. In the SWM, participants were presented with several closed colored boxes and were instructed to search for a small blue token that was hidden in one of the closed boxes. All closed boxes contained a blue token only once; that is, participants had to remember in which boxes they had already found a blue token and in which they had not. Looking inside a closed box that had previously contained a blue token was considered an error (a “between error”). Looking inside a closed box twice within the same search was also considered an error (a “within error”). As outcome variables, we used the following:

(i) Between errors 12 boxes: The number of times the subject revisited a box in which a token had previously been found. Calculated across all trials with 12 tokens only.
(ii) Between errors four boxes: The number of times a subject revisited a box in which a token had previously been found. Calculated across all trials with four tokens only.
(iii) Between errors six boxes: The number of times the subject revisited a box in which a token had previously been found. Calculated across all trials with six tokens only.
(iv) Between errors eight boxes: The number of times the subject revisited a box in which a token had previously been found. Calculated across all trials with eight tokens only.
(v) Between Errors: The number of times the subject incorrectly revisited a box in which a token had previously been found. Calculated across all assessed four-, six- and eight-token trials.
(xvi) Strategy (six and eight boxes): The number of times a subject began a new search pattern from the same box they had started with previously. If they always began a search from the same starting point, we inferred that the subject was employing a planned strategy for finding the tokens. Therefore, a low score indicated high strategy use (1 = they always began the search from the same box); a high score indicated that they began their searches from many different boxes. Calculated across assessed trials with six tokens or eight tokens.

#### Risk-taking behavior

We assessed risk-taking behavior associated with biological and non-biological stimuli using two tasks. Participants were administered with a well-known computerized test investigating risk propensity associated with non-biological stimuli, the Balloon Analogue Risk Task (BART)^26^. They were also tested with a recently modified version of it developed in our lab^41^, the Body Analogue Risk Task (BoART), investigating risk propensity associated with biological stimuli. In the original BART, a balloon was placed at the center of the screen, along with a balloon pump, a button labeled “Collect $$$,” a permanent display labeled “Total Earned” indicating the money earned and a second display listing the money earned on the last balloon, labeled the “Last Balloon.” The participant was asked to click on the pump. Each pump caused a size increase in the balloon (about 0.125 in. [0.3 cm] in all directions), accompanied by a pump sound effect. Additionally, 5 cents were banked in a temporary reserve (not indicated to the subject). When a balloon was pumped to its explosion point, a “pop” sound effect was generated, causing the loss of all banked money. The subjects were instructed that they could stop pumping and click the “Collect $$$” button at any time, transferring all temporary money to the permanent bank. A slot machine payoff sound effect played as the subject clicked on the “Collect $$$” button. After the explosion or the money collection, the balloon disappeared, and a new balloon appeared until a total of 90 balloons had been used/shown. The maximum number of pumps allowed for a single trial was pseudo-randomly chosen within a range that was determined by the balloon’s color (orange life range = 1–8 pumps, yellow life range = 1–32 pumps, blue life range = 1–128 pumps). The three different colors had the purpose of generating an experience-based risk-taking task. Stimuli were presented over three experimental blocks: Block 1 contained the three balloon variants, Block 2 contained the orange and yellow balloons, and Block 3 contained the yellow and blue balloons. Participants were not instructed regarding this difference. Rather, they learned during the task that stimuli had different chances of explosion depending on their colors. Such a learning effect would lead participants to increase their risk-taking behavior over the three blocks linearly. Typically, participants pumped more during the last experimental block when only blue balloons were present, having learned that those had the lowest probability of exploding compared to other balloon colors.

In the modified version of the BART, we replaced the balloon with a body silhouette. As in the case of balloons, each pump increased the stimulus size (about 0.125 in. [0.3 cm] in all directions), and was accompanied by a pump sound effect. Importantly, the human configuration of the silhouette remained plausible throughout inflation. The experimental design was identical to that of the original BART for both the balloon and body tasks. Importantly, we have previously demonstrated that although scores from the two versions of the task were highly correlated, the body task also correlated with core aspects of bodily self-awareness, such as interoception^41^. Each participant performed the two tasks in a randomized order within the group. As outcome variables, we used the following:

(i) Adjusted value: defined as the average number of pumps on the blue stimuli excluding those that exploded (i.e., the average number of pumps on each balloon or body prior to money collection). This exclusion was because the number of pumps was necessarily constrained on the stimuli that exploded, thereby limiting between-subject variability in the absolute averages^26^. As the blue stimuli allowed the widest range in the possible number of pumps and therefore were likely to capture the highest amount of individual variability in task performance, the adjusted number of pumps on these stimuli across blocks served as our primary dependent measure (*risk index*^26,29^).
(ii) Reaction times calculated across all unexploded blue stimuli.

## Statistical analyses

### Data preprocessing

As a first step, we standardized the OC and non-OC participants’ test scores based on the control group’s performance. Values from the two groups of prisoners were transformed in *z*-score according to the mean and standard deviation calculated for each test using the control group’s scores. This allowed us to compare different tests and to gauge cognitive deficits (scores above or below two standard deviations from the control group’s mean)^16,17^.

### Feature selection and data analyses

According to the experimental design, each test had several outcome variables (e.g., reaction times, accuracy, percentages). For this reason, on each test, we applied a data-driven feature selection technique, which is widely used in neuroscience^42–44^, to produce a small number of features for efficient classification or regression and to reduce overfitting and increase the generalization performance of the model. Data were analyzed with SPSS 20 (Statistical Package for Social Science, Windows version, Chicago, Illinois). We used a partial least squares regression (PLS) method to select a subset of key informative behavioral features (one for a cognitive test) based on the resulting variable importance in the projection (VIP) value, to be included in the final regression model. PLS selects the key informative variables by optimizing the model’s performance and has therefore been used to explore possible biomarkers in medicine^45,46^. Furthermore, this data-driven selection of one predictor for cognitive test reduced the risk of multicollinearity that could have occurred if several measures of the same test had been introduced as predictors in the subsequent regression model. However, for the sake of completeness, we have also provided the univariate test results for each variable in the Supplementary Table S1.

Lastly, we used binary logistic regression to determine domains associated with group membership (OC, non-OC) from a set of six predictor variables. Multivariable methods have become routine in statistical analyses appearing in medical literature. A regression model serves two purposes: (i) it can predict the outcome variable for new values of the predictor variables, and (ii) it can help answer questions about the most important predictors of the OC cognitive profile, because the coefficient of each predictor variable explicitly describes the relative contribution of that variable to the outcome variable, automatically controlling for the influences of the other predictor variables. As the two groups of prisoners differed in their duration of detention, we also inserted it as a covariate in the regression model. As we had no *a priori* hypothesis on the role that different variables play in the prediction of the OC membership, we used a forward stepwise (likelihood ratio) method. To deal with potential outliers influencing the regression model, we also performed a case-wise diagnostic on Studentized residuals (i.e., the quotients resulting from the division of a residual by an estimate of its standard deviation), and cases with Studentized residuals >2 were excluded. We found one influential case within the non-OC group that was excluded from the subsequent analyses.

## Supplementary information

Supplementary information.

## Data Availability

The data that support the findings of this study are available from the corresponding author upon reasonable request.
