# Supplementary material for: Pathological risk-propensity typifies Mafia members’ cognitive profile
Source: Sci Rep. 2020 May 22;10:8559. doi: 10.1038/s41598-020-65486-z (PMC7244520; doi:10.1038/s41598-020-65486-z)
Supplement: Supplementary file 1 — Supplementary information. [file 41598_2020_65486_MOESM1_ESM.pdf]

Supplementary Information for:

Pathological risk-propensity typifies Mafia members' cognitive profile.

G. Salvato[1,2,3], M.L. Fiorina[1], G. De Maio[1], E. Francescon[1], D. Ovadia[1,3], L. Bernardinelli[1], A. Santosuosso[4], E. Paulesu[5,6], G. Bottini[1,2,3,]

[1] Department of Brain and Behavioral Sciences, University of Pavia, Pavia, Italy,
[2] Cognitive Neuropsychology Centre, ASST "Grande Ospedale Metropolitano" Niguarda, Milano, Italy,
[3] NeuroMi, Milan Centre for Neuroscience, Milan, Italy.
[4] European Centre for Law, Science and New Technologies (ECLT), University of Pavia, Pavia, Italy
[5] Psychology Department and NeuroMI-Milan Center for Neuroscience, University of Milano-Bicocca, Milan, Italy.
[6] fMRI Unit, IRCCS Istituto Ortopedico Galeazzi, Milan, Italy.

Dependent Variable	Chi-Square	df	p-value
Cognitive Flexibility			
* MTTICMD	1.010	1	.315
MTTLMD	1.010	1	.315
MTTMTCMD	.000	1	1.000
MTTTIC	.000	1	1.000
Planning			
OTSMDLFC	1.382	1	.240
*OTSPSFC	5.263	1	.022
Sustained Attention			
RVPA	.000	1	1.000
RVPMDL	.444	1	.505
*RVPPFA	.709	1	.400
Working Memory			
SWMBE12	.000	1	.984
SWMBE4	1.412	1	.235
SWMBE468	.154	1	.695
* SWMBE6	4.332	1	.037
SWMBE8	.709	1	.400
SWMS	.000	1	1.000
Risk-taking (BoART)			
* BODY PGA	1.895	1	.169
BODY TA	.709	1	.400
Risk-taking (BART)			
* BALLOON PGA	1.515	1	.281
BALLOON TA	.444	1	.505

Supplementary Table S1. The table shows the between-groups differences for all the variables for each cognitive test. Each variable was binary, coded as 0=no deficit (scores ±2 standard deviations from the mean) and 1=deficit (scores ±2 standard deviations from the mean). The asterisk indicates the predictors that have been inserted in the final model according to the PLS analysis.

Detailed description for each measure (see also the Materials and methods section in the manuscript):

MTTICMD: Multitasking incongruency cost (median): The difference between the median latency of response (from stimulus appearance to button press) on the trials that were congruent versus the incongruent trials. This was calculated by subtracting the median congruent-trial latency (in ms) from the median incongruent-trial latency. A positive score indicates that the subject was faster on congruent trials, and a negative score indicates that the subject was faster on incongruent trials. A higher incongruency cost indicates that the subject took longer to process conflicting information.

MTTMTCMD: Multitasking cost (median): The difference between the median latency of response (from stimulus appearance to button press) during assessed blocks in which both rules were used, versus assessed blocks in which only a single rule was used. Calculated by subtracting the median latency of response during single-task blocks from the median latency of response during multitasking

blocks. A positive score indicates that the subject responded more slowly during multitasking blocks and indicates a higher cost of managing multiple sources of information.

MTTLMD: Reaction latency (median): The median latency of response (from stimulus appearance to button press). Calculated across all correct trials.

MTTTIC: Total incorrect: The number of trials for which the outcome was an incorrect response (subject pressed the wrong button within the response window). Calculated across all assessed trials.

OTSMDLFC: Median Latency to First Choice: The median latency, measured from the appearance of the stocking balls until the subject made the first box choice. Calculated across all assessed trials where the subject's first response was correct.

OTSPSFC: Problems solved on the first choice: The total number of assessed trials where the subject chose the correct answer on their first attempt. Calculated across all assessed trials.

RVPA: A' (A prime) is the signal detection measure of a subject's sensitivity to the target sequence (a string of three numbers), regardless of response tendency (the expected range is 0.00 to 1.00; bad to good). In essence, this metric is a measure of how good the subject is at detecting target sequences.

RVPMDL: Median response latency: The median response latency on trials where the subject responded correctly, calculated across all assessed trials.

RVPPFA: Probability of false alarm: The number of sequence presentations that were false alarms divided by the number of sequence presentations that were false alarms plus the number of sequence presentations that were correct rejections: (false alarms ÷ (false alarms + correct rejections)).

SWMBE12: Between errors 12 boxes: The number of times the subject revisited a box in which a token had previously been found. Calculated across all trials with 12 tokens only.

SWMBE4: Between errors four boxes: The number of times a subject revisited a box in which a token had previously been found. Calculated across all trials with four tokens only.

SWMBE6: Between errors six boxes: The number of times the subject revisited a box in which a token had previously been found. Calculated across all trials with six tokens only.

SWMBE8: Between errors eight boxes: The number of times the subject revisited a box in which a token had previously been found. Calculated across all trials with eight tokens only.

SWMBE468: Between Errors: The number of times the subject incorrectly revisited a box in which a token had previously been found. Calculated across all assessed four-, six- and eight-token trials.

SWMS: Strategy (six and eight boxes): The number of times a subject began a new search pattern from the same box they had started with previously. If they always began a search from the same starting point, we inferred that the subject was employing a planned strategy for finding the tokens. Therefore, a low score indicated high strategy use (1 = they always began the search from the same box); a high score indicated that they began their searches from many different boxes. Calculated across assessed trials with six tokens or eight tokens.

BODY PGA: Adjusted value: defined as the average number of pumps on the blue body stimuli excluding those that exploded (i.e., the average number of pumps on each balloon or body prior to money collection).

BODY TA: Reaction times calculated across all unexploded blue body stimuli.

BALLOON PGA: Adjusted value: defined as the average number of pumps on the blue balloon stimuli excluding those that exploded (i.e., the average number of pumps on each balloon or body prior to money collection).

BALLOON TA: Reaction times calculated across all unexploded blue balloon stimuli.
